# Cost-Effectiveness of a Lifestyle and Behavioral Care Model Targeting Cardiometabolic Disease Progression

**DOI:** 10.3390/ijerph23040526

**Published:** 2026-04-18

**Authors:** Michelle Alencar, Rachel Sauls, Justin Whetten

**Affiliations:** 1 Department of Kinesiology, California State University Long Beach, Long Beach, CA 90840, USA; 2 InHealth Medical Services Inc., Los Angeles, CA 90067, USA; rsauls@inhealthonline.com; 3 Department of Nutrition, Gillings School of Global Public Health, University of North Carolina at Chapel Hill, Chapel Hill, NC 27516, USA; 4 Medical Economics, University of New Mexico Health System, Albuquerque, NM 87106, USA; jswhetten@unmmg.org

**Keywords:** behavioral model, return on investment, chronic care, health and wellness coaching, monetary reimbursement

## Abstract

**Highlights:**

**Public health relevance—How does this work relate to a public health issue?**

- Chronic cardiometabolic diseases—major drivers of U.S. morbidity, mortality, and healthcare spending—are directly addressed through a scalable telehealth behavioral care model.
- The study evaluates an intervention that targets lifestyle-related risk factors, a central population-level lever for reducing chronic disease burden.

**Public health significance—Why is this work of significance to public health?**

- The program demonstrates substantial cost savings ($6403 per member; $28.6 M population savings) and health gains (0.1 QALY), showing that behavioral interventions can yield meaningful, measurable health improvements.
- The high ROI (6.53) provides rare evidence that prevention-focused behavioral care models can be both clinically effective and economically sustainable at scale.

**Public health implications—What are the key implications or messages for practitioners, policy makers and/or researchers in public health?**

- Employers and health systems can integrate telehealth-enabled behavioral care into benefit designs to reduce long-term healthcare costs while improving employee health outcomes.
- Policymakers and researchers may use the provided modeling framework as a reproducible method for evaluating cost-effectiveness and ROI of lifestyle and chronic disease interventions.

**Abstract:**

Chronic diseases drive healthcare costs, and employers seek scalable strategies to improve health outcomes and control expenses. Telehealth behavioral care shows promise for managing chronic conditions, but its long-term economic value in employer populations is still unclear. We assessed the cost-effectiveness and ROI of a behavioral care (LBC) model using a Markov model in a custom analytic tool. The model simulated disease progression, healthcare utilization, and QALYs over five years from the employer perspective. Transition probabilities, costs, and mortality risks were obtained from the InHealth program, national sources, and published literature. Employees in the behavioral care model were compared with a control group receiving usual care. Among 4461 employees aged 40, intervention participants had five-year costs of $41,431, versus $47,834 for controls, saving $6403 per member and $28.6 million overall. Treated members gained 4.7 QALYs compared to 4.6 in controls, equivalent to 36.5 extra days of full health. The program had a ROI of 6.53, showing significant cost savings. Telehealth behavioral care is a cost-effective way to improve health outcomes and provide financial benefits to employers. These results support incorporating behavioral care into value-based benefits and highlight potential long-term savings through prevention and management of lifestyle-related chronic diseases.

## 1. Introduction

Cardiometabolic chronic diseases such as diabetes, hypertension, and cardiovascular conditions continue to impose a substantial and growing burden on healthcare systems worldwide [1]. The incidence of lifestyle-related chronic conditions (e.g., diabetes, cardiovascular disease, and obesity) continues to rise globally, contributing substantially to healthcare utilization and costs [1,2]. These conditions typically require continuous monitoring, medication management, and frequent clinical encounters, placing increasing strain on health systems [2]. Because of this growing burden, cost-effective interventions are urgently needed to support patients outside traditional care settings [3]. Telehealth and similar digital health programs have emerged as one such solution, offering expanded access to care, improved continuity of care, and better health outcomes [4,5,6,7,8]. As digital health solution adoption accelerates, evaluating the economic impact of these programs has become increasingly important to ensure their long-term sustainability [6,7]. Most existing evaluations focus primarily on clinical outcomes, short-term utilization changes, or patient satisfaction [7,9]. Far fewer studies provide rigorous, transparent, and reproducible economic analyses, particularly within large, real-world populations or programs targeting multiple cardiometabolic conditions simultaneously.

Metrics such as return on investment (ROI) can help fill this gap by providing decision-makers with clear insights into financial value [9,10]. Unlike a traditional budget impact analysis (BIA), which focuses on short-term, year-over-year changes in expenditures to inform affordability and budgeting decisions, ROI quantifies the financial return relative to the dollars invested, capturing downstream cost offsets from risk reduction or avoided medical events [11]. In general, a higher ROI indicates greater financial benefit relative to the investment, making it a practical tool for employers and health-system leaders assessing long-term value [10,11,12]. ROI has gained traction as a key metric for assessing health programs, especially among healthcare systems, employers, and policymakers responsible for allocating limited resources efficiently [9]. Unlike traditional cost-effectiveness measures, such as incremental cost-effectiveness ratios (ICERs) or quality-adjusted life years (QALYs), ROI offers a financially intuitive perspective that resonates with operational and administrative stakeholders [12,13]. Systematic reviews have demonstrated that public health interventions often generate substantial economic returns, with some local programs reporting a median ROI of 4.1:1 [9,11]. Despite these findings, ROI analyses are often program-specific, vary widely in methodology, and frequently lack the transparency needed for replication or comparison across interventions [9,14]. Moreover, few studies apply ROI frameworks to comprehensive telehealth or behavioral support model programs targeting multiple cardiometabolic chronic diseases simultaneously, representing a critical area where decision-makers require better evidence [9]. This growing body of evidence highlights ROI as a valuable complement to traditional economic evaluation methods, providing insight into both health benefits and financial viability.

To rigorously assess the long-term effects of chronic disease interventions, health economists frequently employ modeling approaches such as Markov models [10,15]. These models simulate disease progression by allowing individuals to transition between defined health states over time, each associated with distinct costs and utility values [14,15]. Markov modeling is particularly well-suited to chronic diseases, where outcomes accumulate gradually, and clinical trajectories can vary widely [14,16,17,18]. Both deterministic and probabilistic simulations can be incorporated to account for parameter uncertainty and enhance analytic robustness [19,20]. While Markov models are widely used in chronic disease research [21,22], they are rarely applied to employer-based, telehealth-enabled behavioral care models targeting lifestyle-related chronic disease. There is a lack of standardized modeling tools that integrate real-world program data with evidence-based transition probabilities to estimate both cost-effectiveness and ROI over multi-year horizons.

The InHealth program is a telehealth-enabled lifestyle and behavioral care (LBC) model designed to support individuals with multiple chronic conditions by combining remote monitoring, structured behavioral support, and ongoing engagement [23,24]. This modality was selected because prior clinical work, including trials directly comparing video-based and in-person coaching models, demonstrates that telehealth can enhance personalization, extend reach, and facilitate more meaningful interactions (such as allowing participants to show their home environment or food choices in real time), offering advantages beyond coaching alone and supporting a more comprehensive, integrated approach to chronic-care management [23,24].

To address the existing evidence gap, this study evaluates the economic and clinical impact of the InHealth LBC program within a large national employer population. Using a multi-year economic modeling framework informed by real-world program data and published evidence, we estimate direct medical costs, quality-adjusted life years (QALYs), and return on investment (ROI). Evaluating the financial and clinical value of the InHealth LBC program within a large national employer context helps address this evidence gap. The findings offer important implications for employers and health systems, aiming to manage the costs of chronic disease.

## 2. Materials and Methods

### 2.1. Model Overview

A state-transition Markov model was developed using a custom simulation tool to evaluate the cost-effectiveness and return on investment (ROI) of the InHealth LBC model [14,23]. A cohort-based state-transition Markov model was selected due to its suitability for representing chronic disease progression over time, where individuals transition between defined health states in discrete intervals. The model assumes a memoryless process, whereby transition probabilities depend only on the current health state. The model simulates annual transitions among chronic disease states and estimates direct medical costs and health outcomes, expressed as quality-adjusted life years (QALYs) over specified time horizons.

### 2.2. Model Structure

**Figure 1:**
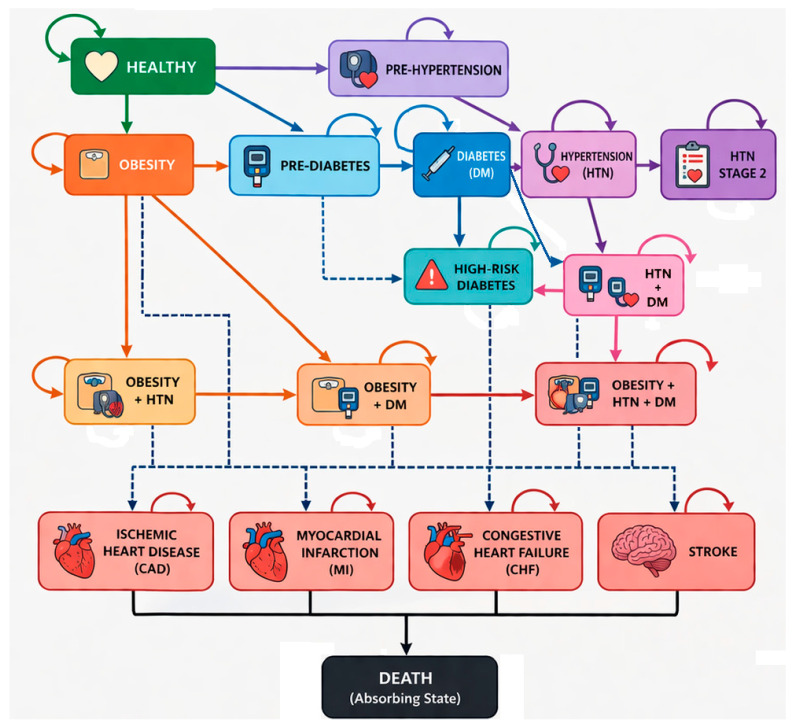
Markov model structure of health state transitions. Arrows indicate allowable transitions between health states in each cycle; loops to the same state represent remaining in that state.

The model includes chronic disease states representing major conditions commonly targeted by health and wellness coaching: diabetes, hypertension, cardiovascular disease, cancers, respiratory illness, and mental health disorders. Health states were modeled as mutually exclusive, with comorbid conditions represented through explicit composite health states (e.g., diabetes with hypertension), allowing for parallel risk adjustment of transition probabilities, costs, and outcomes. Each state is associated with a specific annual cost and utility (QALY weight). Most conditions are modeled as progressive and irreversible, except for obesity, diabetes, and hypertension, which may improve in response to behavioral modification interventions, as seen in Figure 1.

Annual transitions between states occur in discrete one-year cycles. Transition probabilities reflect the likelihood of developing new conditions, progressing within disease states, experiencing acute events (e.g., myocardial infarction or stroke), or death. Mortality risk is modeled using age-specific mortality rates from CDC life tables [25].

### 2.3. Data Inputs and Parameter Sources

The decision-analytic model was developed in Microsoft Excel (Microsoft Corporation, Redmond, WA, USA; Version 365), allowing for flexible adjustment of inputs and scenario testing. Model inputs can be adjusted based on the population/employer needs and baseline starting scenario. These inputs include: starting age for receiving health and wellness coaching at InHealth Medical Services (e.g., 40 years), time horizon (5 years, 10 years, or lifetime), analytic perspective (e.g., employer or health plan), population health distribution at baseline (e.g., diabetes, hypertension, obesity), engagement tier (e.g., number of coaching sessions, incentives), and annual program cost (default: $720).

Model parameters were obtained from program-specific data, published literature, and publicly available datasets. Transition probabilities were informed by epidemiologic studies and, where necessary, calibrated to reflect observed baseline population risk. Cost inputs were obtained from published cost-of-illness studies and adjusted to 2023 USD using the medical component of the consumer price index. Mortality inputs were derived from CDC life tables [25]. Table 1 summarizes key model parameters, their sources, and default values.

The model supports both deterministic simulations (using mean values) and probabilistic simulations (using sampled distributions) as seen in Table 2.

Health-state utility values and disutility weights for acute events were drawn from peer-reviewed studies on chronic disease and health utility measurement. QALYs were calculated as the product of health-state utility and time spent in each state.

### 2.4. Study Population

The modeled intervention cohort consisted of employees enrolled in the InHealth LBC programs, which provide structured interventions targeting lifestyle-related conditions (e.g., weight management, blood pressure control). Baseline age was set at 40 years as a representative working adult population, though the model can be adjusted for other ages. Demographic and clinical characteristics were drawn from program enrollment data: for example, the cohort was 55% female, had a mean BMI of 29 kg/m^2^, and 23.5% had diagnosed hypertension at baseline. Baseline prevalence of other chronic conditions was incorporated to reflect the underlying risk profile of the population (see Table 1).

### 2.5. Intervention (InHealth LBC Program)

Participants received structured lifestyle and behavioral care aimed at improving diet, physical activity, medication adherence, weight management, and other lifestyle behaviors. Coaching frequency and intensity varied by engagement tier and followed standardized program protocols. The intervention effect was modeled as a relative reduction in disease progression and incidence risks, informed by published lifestyle intervention studies and program-specific outcomes data [26,32].

### 2.6. Control Group

The control group followed the baseline assumptions embedded in the tool. Specifically, the control group reflected standard care in the U.S. healthcare system for managing chronic diseases, including routine primary care visits, medication management, and disease monitoring. Transition probabilities captured typical chronic disease progression and healthcare utilization patterns in the absence of structured lifestyle coaching. Both intervention and control groups were assumed to receive standard medical care for chronic conditions, ensuring comparable treatment pathways aside from the coaching component. The control group is a modeled comparator based on national averages and baseline assumptions embedded in the tool; it does not represent an observed, real-world cohort. All outcomes for the control group, including costs and QALYs, are therefore conditional on these assumptions.

### 2.7. Analytic Framework

For each cycle, the model estimated:

- Direct medical costs;
- Program costs (intervention only);
- QALYs;
- Healthcare utilization differences between groups.

Total expected cost and QALYs were accumulated over the time horizon accounting for a discounting rate of 3%.

### 2.8. Return on Investment (ROI)

ROI was calculated as:

$$ROI=\frac{Cost\;Savings}{Program\;Costs}$$

where cost savings represent the difference in total direct medical costs between the control and intervention groups.

### 2.9. Model Outputs

Outputs included:

- Annual and cumulative cost differences;
- QALYs gained;
- ROI estimates;
- Per-member and population-level results;
- Scenario-specific outputs based on adjustable model inputs.

Interactive visualizations are available to display temporal trends in costs, QALYs, and ROI.

### 2.10. Validation and Assumptions

Model validation occurred at two levels:

1. Internal validation—using historical InHealth program data to confirm expected changes in risk factors and utilization.
2. External validation—comparing cost and QALY outputs to published cost-effectiveness studies of similar interventions.

Key assumptions included:

- Identical treatment pathways for intervention and control groups outside of coaching participation;
- Progressive disease states, except for obesity, diabetes, and hypertension;
- A 3% annual discount rate for both costs and QALYs was applied in the base case, with alternative scenarios explored in sensitivity analyses. Full adherence to coaching among participants classified as “engaged”.

A key limitation of this study is that the control group is not composed of real-world patients but is instead modeled from national averages. Consequently, cost savings and QALY gains are dependent on the assumptions built into the model and may not fully reflect outcomes in a true usual-care population.

### 2.11. Sensitivity Analyses

The model enables deterministic sensitivity analysis by modifying key parameters (e.g., program cost, engagement level, relative risk reductions). Probabilistic sensitivity analysis (PSA) was conducted using Monte Carlo simulation, with 10,000 iterations. Parameter uncertainty was modeled using appropriate distributions (beta for probabilities and utilities, gamma for costs, and lognormal for relative risks), based on standard health economic practices.

## 3. Results

Under default settings (age 40, employer perspective, 5-year horizon, 12 sessions, $720 annual cost), the model yields:

- Cost per treated member: $41,431;
- Cost per control member: $47,834;
- QALYs: 4.7 (inHealth) vs. 4.6 (Control);
- Savings: $6403 and 0.1 QALY (≈36.5 days of full health);
- ROI: 6.53.

The InHealth Cost-Effectiveness and ROI Tool estimates the program’s financial and clinical impact by comparing projected medical spending and quality-adjusted life years (QALYs) between treated and untreated employees. The model shows that participants incur $41,431 in healthcare costs versus $47,834 for controls, alongside a modest quality-of-life improvement (4.7 vs. 4.6 QALYs, or roughly 36.5 additional days of full health). These differences translate into $6403 in net savings per participant, with an ROI of 6.53, indicating that the program more than pays for itself over time. Applied to a population of 4461 employees, the analysis suggests the InHealth lifestyle-behavior-change program is both cost-saving and clinically beneficial, reducing expected healthcare expenditures while producing measurable gains in health outcomes.

### 3.1. Cost Savings

Participants receiving the LBC intervention incurred an average total cost of $41,431 over five years, compared to $47,834 for those in the control group. This represents a net saving of $6403 per member. The primary drivers of these reductions were medication and healthcare service utilization.

- At the population level, the program generated total net savings of $28,564,167. This was calculated using a modeled population of 4461 participants, with an average per-person net savings of $6403. Total savings were broken down as follows:
- Medication savings: $9,880,784;
- Healthcare services savings: $23,055,163;
- Less program costs of: $4,371,780.

*Calculation*: *Total savings* = (*Medication savings* + *Healthcare services savings*) − *Program costs* = $28,564,167.

### 3.2. Health Outcomes

The intervention produced modest but meaningful improvements in health status. Treated employees accrued 4.7 QALYs, compared with 4.6 QALYs in the control group, representing an incremental gain of 0.1 QALY (≈36.5 additional days in perfect health). These gains reflect reductions in chronic disease progression and associated complications, underscoring the clinical value of coaching interventions.

### 3.3. Return on Investment

**Figure 2:**
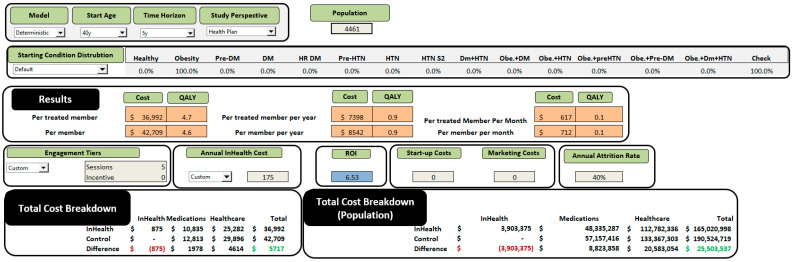
ROI tool with cost comparison for InHealth LBC Employer Group. Figures were created using Microsoft Excel (Microsoft Corporation, Redmond, WA, USA; Version 365).

The calculated ROI was 6.53, meaning every dollar invested in health coaching returned $6.53 in avoided healthcare costs. This high ROI reflects the compounding effect of early modification of risk factors and the prevention of costly downstream events, as seen in Table 3 and Figure 2.

### 3.4. Sensitivity Analyses Results

Deterministic sensitivity analyses indicated that ROI remained positive under a range of program costs, engagement levels, and effect sizes. Probabilistic sensitivity analysis (10,000 iterations) produced a mean ROI of 6.3 (95% CI: 4.8–7.9), confirming the robustness of the economic impact.

Key drivers of variation included program engagement and intervention effect on chronic disease progression.

### 3.5. Model Validation

Internal validation confirmed that simulated changes in risk factors and utilization patterns aligned with historical InHealth program data. External validation against published studies showed comparable QALY gains and cost savings, supporting the model’s reliability.

## 4. Discussion

The findings from this analysis underscore the economic and clinical value of the behavioral care model as a strategic intervention for employers. Over a five-year horizon, the InHealth program demonstrated a $28.6 million reduction in total healthcare costs for a population of 4461 employees, driven by lower medication and healthcare service expenditures. The calculated ROI of 6.53 indicates that every dollar invested in coaching yields more than six dollars in avoided costs, a return that exceeds typical benchmarks for employer-sponsored health initiatives.

The findings are consistent with a growing body of literature supporting the effectiveness of behavior change interventions in managing chronic conditions [28,34]. Prior studies have shown that coaching improves self-management behaviors, increases adherence to evidence-based care, and reduces avoidable healthcare utilization [28]. While our study reports population-level cost savings and ROI, and not cost per QALY, these findings align conceptually with international evidence on the value of lifestyle and behavioral interventions [34]. For example, a randomized controlled trial of tele-based health coaching for patients with diabetes and cardiovascular disease in the UK reported cost-effectiveness ratios (ICER) as low as €20,000 per QALY. Although ROI and ICER are different metrics, both indicate economic value and efficiency of lifestyle interventions. Similarly, U.S. evaluations have found reductions in inpatient utilization of nearly 50% and outpatient savings of approximately $286 per member per month, translating to overall cost reductions of $412 per person per month [34]. These findings support the mechanisms observed in the present study: improved risk factor control, reduced disease progression, and lower incidence of costly complications.

Beyond cost savings, the modest improvement in QALYs observed in this analysis (4.7 vs. 4.6) represents approximately 36.5 additional days of full health per participant, reflecting clinically meaningful gains in health status [27]. Importantly, these gains are conditional on model assumptions, including the use of a modeled control group based on national averages rather than a real-world cohort. The benefits of behavioral interventions compound over time, as prevention of chronic disease progression and associated complications leads to exponential cost avoidance in later years [14,28,30].

The implications for employers and health systems are substantial. Rising chronic disease prevalence and healthcare costs that outpace wage growth create an urgent need for interventions that improve population health while reducing expenditures. Behavioral interventions, such as the InHealth LBC program, which include health coaching, represent a flexible, evidence-based approach that can be integrated into broader population health strategies, including digital health tools, remote monitoring, and incentive programs [23,24]. These programs are grounded in well-established theories of health behavior change (e.g., social cognitive theory, self-determination theory, and the transtheoretical model) [29,33], which emphasize that sustained behavior change occurs through ongoing support, goal-setting, feedback, and repeated opportunities to practice healthier choices [35]. High engagement rates further strengthen the pathway to behavior change by increasing exposure to these evidence-based mechanisms, maximizing the likelihood of sustained risk reduction.

Despite these strengths, several limitations should be noted. Most importantly, the control group is modeled using national averages and assumptions embedded in the tool, and does not reflect real-world usual-care outcomes. As a result, all cost savings and QALY gains are conditional on these assumptions. Future studies should evaluate the durability of observed benefits beyond five years, examine heterogeneity across demographic subgroups, risk profiles, and engagement levels, and incorporate real-world claims data to strengthen generalizability. In terms of generalizability, the findings of this study should be assessed in light of the study population being specific to a working-age employee population (age 40, 55% female, BMI 29), and more research is needed to assess other working age groups (e.g., above 18, above 65 not retired yet). Additionally, research should explore the combined effects of coaching with complementary interventions, such as digital health applications, social determinants of health supports, or employer-based incentives, to identify synergistic opportunities that maximize cost savings and health impact. Furthermore, this study evaluates a program associated with the authors’ affiliated organization, which may introduce potential bias. Although model development and analytic oversight were conducted by an independent co-author, and inputs were informed by published and public data sources, independent external validation of the model and findings would further strengthen confidence in the results

## 5. Conclusions

Lifestyle and behavioral care that includes health coaching is a high-value, evidence-based strategy for improving employee health, reducing healthcare expenditures, and generating strong financial returns for employers. In this analysis, the InHealth program produced $28.6 million in cost savings and achieved an ROI of 6.53 over five years, driven by sustained reductions in medication use, healthcare service utilization, and chronic disease progression. Participants experienced measurable improvements in health outcomes, including an average gain of 0.1 QALY, reflecting enhanced engagement, adherence, and self-management.

These findings align with national evidence showing that employer-based programs focusing on behavior change for chronic diseases reduce healthcare spending and routinely deliver 3–6× returns on investment. High engagement indicators, such as activation, program completion, goal attainment, and clinically significant risk improvement, further support the effectiveness of coaching as a behavior-change strategy.

Collectively, the evidence indicates that a telehealth-enabled behavioral care model is a strategic, sustainable investment for employers seeking to improve workforce health and control long-term medical costs. Integrating coaching into broader wellness and population health initiatives may yield even greater benefits, positioning it as a key component of modern value-based benefit design.

## Figures and Tables

**Table 1:** Key model parameters and their respective sources and default values in the model.

Health State	Annual Cost (USD)	Cost Distribution	Utility	Utility Distribution	Source
Healthy	$0	Gamma(100, 0)	1	Beta(17.1, 0.9)	[26,27,28,29,30,31,32,33,34]
Obesity	$1200	Gamma(100, 12)	0.88	Beta(880, 120)	[26,27,28,29,30,31,32,33,34]
Pre-Diabetes	$3000	Gamma(100, 30)	0.87	Beta(87, 13)	[26,27,28,29,30,31,32,33,34]
Pre-Hypertension	$1000	Gamma(100, 10)	0.86	Beta(86, 14)	[26,27,28,29,30,31,32,33,34]
Hypertension (HTN)	$2500	Gamma(100, 25)	0.85	Beta(850, 150)	[26,27,28,29,30,31,32,33,34]
HTN Stage 2	$4000	Gamma(100, 40)	0.8	Beta(80, 20)	[26,27,28,29,30,31,32,33,34]
Diabetes (DM)	$9500	Gamma(100, 95)	0.7	Beta(70, 30)	[26,27,28,29,30,31,32,33,34]
Ischemic Heart Disease (CAD)	$12,000	Gamma(100, 120)	0.78	Beta(780, 220)	[26,27,28,29,30,31,32,33,34]
Myocardial Infarction (MI)	$20,000	Gamma(100, 200)	0.75	Beta(75, 25)	[26,27,28,29,30,31,32,33,34]
Congestive Heart Failure (CHF)	$14,000	Gamma(100, 140)	0.68	Beta(68, 32)	[26,27,28,29,30,31,32,33,34]
Stroke	$12,000	Gamma(100, 120)	0.6	Beta(60, 40)	[26,27,28,29,30,31,32,33,34]
DM + HTN	$11,000	Gamma(100, 110)	0.6	Beta(60, 40)	[26,27,28,29,30,31,32,33,34]
Obesity + HTN	$3200	Gamma(100, 32)	0.75	Beta(75, 25)	[26,27,28,29,30,31,32,33,34]
Obesity + DM	$10,700	Gamma(100, 107)	0.62	Beta(62, 38)	[26,27,28,29,30,31,32,33,34]
Obesity + HTN + DM	$12,500	Gamma(100, 125)	0.55	Beta(55, 45)	[26,27,28,29,30,31,32,33,34]
High-Risk Diabetes	$14,500	Gamma(100, 145)	0.55	Beta(55, 45)	[26,27,28,29,30,31,32,33,34]

**Table 2:** Deterministic simulation and probabilistic simulation mechanisms.

Transition	Annual Probability	Distribution	Source
Healthy → Obesity	2.46%	Beta(50, 950)	[27,30]
Healthy → Pre-Diabetes	2.24%	Beta(20, 980)	[27,30,33]
Healthy → Pre-HTN	0.37%	Beta(30, 970)	[27,30]
Obesity → Pre-DM	3.81%	Beta(80, 920)	[27,30,33]
Obesity → DM	1.64%	Beta(30, 970)	[27,30,33]
Pre-DM → DM	1.12%	Beta(70, 930)	[27,30,33]
Pre-DM → High-Risk DM	0.44%	Beta(10, 990)	*Derived [27,30]
Pre-HTN → HTN	0.41%	Beta(50, 950)	[27,30]
HTN → HTN Stage 2	0.25%	Beta(20, 980)	*Derived [27,30]
DM → High-Risk DM	1.31%	Beta(30, 970)	[27,30]
DM + HTN → High-Risk DM	1.57%	Beta(40, 960)	*Derived [27,29,30]
Any → CAD	0.16%	Beta(10, 990)	[27,30]
Any → MI	0.10%	Beta(50, 950)	[27,30]
Any → CHF	0.26%	Beta(20, 980)	[27,30]
Any → Stroke	0.20%	Beta(10, 990)	[27,30]
Combined states → higher-risk	0.03–0.05%	Beta	*Calibrated [27,30]
Any → Death	Age-specific	Beta	[32]

* Parameters not directly available in the literature were estimated using proportional scaling, additive cost assumptions, or multiplicative risk adjustments, and calibrated to match observed epidemiologic patterns.

**Table 3:** Cost comparison table (per member and population).

	LBC (Treatment)	Control	Difference
Total cost per member (5 years)	$41,431	$47,834	$6403
Annual cost per member	$8285.76	$9567	$1281
Monthly cost per member	$691	$797	$106
Population total cost	$184,823,517	$213,387,685	$28,564,167

## Data Availability

Data is available upon request due to anonymization of patient data.
